# Fermented lingonberry juice's effects on active MMP‐8 (aMMP‐8), bleeding on probing (BOP), and visible plaque index (VPI) in dental implants—A clinical pilot mouthwash study

**DOI:** 10.1002/cre2.638

**Published:** 2022-07-27

**Authors:** Hanna Lähteenmäki, Taina Tervahartiala, Ismo T. Räisänen, Pirjo Pärnänen, Timo Sorsa

**Affiliations:** ^1^ Department of Oral and Maxillofacial Diseases, Head and Neck Center University of Helsinki and Helsinki University Hospital Helsinki Finland; ^2^ Department of Dental Medicine, Division of Periodontology Karolinska Institutet Huddinge Sweden

**Keywords:** dental implants, lingonberries, mouthwash, *Vaccinium vitis‐idaea*

## Abstract

**Objectives:**

We aimed to study the effects of fermented lingonberry juice (FLJ) as a mouthwash on the levels of active matrix metalloproteinase‐8 (aMMP‐8) in peri‐implant sulcular fluid (PISF), bleeding on probing (BOP), and visible plaque index (VPI). We hypothesized that FLJ rinsing could reduce inflammation (aMMP‐8 and BOP) and microbial load (VPI) in the oral cavity, especially around dental implants.

**Materials and Methods:**

A clinical pilot study was performed using FLJ as a mouthwash. The inclusion criteria were at least one dental implant in the anterior or posterior areas with a screw‐retained crown. Ten participants used 10 ml of mouthwash twice a day for 15 days, and 10 participants served as the control group. Point‐of‐care tests (POCTs) were used to measure aMMP‐8 levels in the PISF, and BOP and VPI were recorded at the beginning of the trial and after 15 and 30 days.

**Results:**

The FLJ mouthwash had a reductive effect on aMMP‐8, VPI, and BOP in the mouthwash group; however, there was no significant difference compared to the control group. The difference in VPI and BOP levels between the groups diminished after the lingonberry regimen ended. The decrease in aMMP‐8 levels appeared to continue even after discontinuation of the mouthwash regimen.

**Conclusion:**

The reduction in the amount of plaque, aMMP‐8, and BOP by FLJ was promising and continuous considering the relatively short study period and sample size. FLJ is a natural and safe supplement for oral and dental implant home care. Further studies are required to verify these promising results.

## 1 INTRODUCTION

### 1.1 Lingonberry

The positive health effects of berries have been studied in various laboratories and clinical trials (Brown et al., 2014; Ek et al., 2006; Heyman‐Lindén et al., 2016; Nile & Park, 2014). Lingonberries (*Vaccinium vitis‐idaea* L.) have a high mineral, vitamin, antioxidant, and polyphenol content. According to certain studies, lingonberry polyphenols appear to have anti‐inflammatory properties (Kivimäki et al., 2014; Nile & Park, 2014; Yahfoufi et al., 2018) and antimicrobial effects (Kylli et al., 2011; Tian et al., 2018). Fermented lingonberry juice (FLJ) inhibits in vitro *Candida glabrata* stress‐ and energy metabolism‐related intracellular protein expression (Pärnänen et al., 2017) and the growth of periodontopathogens *Candida* and *Streptococcus mutans*. Additionally, it reduces dental plaque (visual plaque index, VPI), bleeding on probing (BOP), and active matrix metalloproteinase‐8 (aMMP‐8) levels, as demonstrated in an in vivo human study (Pärnänen et al., 2019). FLJ has also been shown to inhibit the activation of procollagenase‐2 (MMP‐8) by *Candida glabrata* cell wall proteases (Pärnänen et al., 2020). Despite structural modifications following digestion and fermentation, lingonberry extracts retain their biological activity in vitro (Brown et al., 2014).

Previous studies have shown that polyphenols in the high molecular size fractions of crowberry, blackcurrant, bilberry, and lingonberry juices exert an antiaggregation effect on plaque colonizers in vitro (Riihinen et al., 2011). Biofilm formation on dental implant surfaces may be reduced by several treatment modalities (Garaicoa et al., 2020). Chlorhexidine (CHX) mouthwash and gels used in oral home care have been evaluated by several studies (Bescos et al., 2020; Fiorillo, 2019; James et al., 2017); however, a range of negative side effects has been reported, including discoloration of teeth, taste disturbance/alteration, and oral mucosa symptoms such as soreness. Compared to CHX mouthwash, FLJ mouthwash has not been reported to exert these side effects.

There are no previous studies on the effects of lingonberries on dental implants, and because of the versatility and safety of lingonberry polyphenols in reducing oral inflammation and microbial load, FLJ appears to be a promising treatment candidate.

### 1.2 Dental implants, peri‐implant mucositis, and peri‐implantitis

Dental implants are a common treatment for replacing missing teeth. Dental implants require careful home care as infection and inflammation progress faster in dental implants than in natural teeth, and the treatment of dental mucositis is regarded to be more challenging than that of periodontitis (Golub et al., 2020). There is a variety of bacteria and yeasts in the oral cavity that form biofilms on all surfaces in a fluid system, as well as on hard, nonshedding surfaces such as oral implants (Bescos et al., 2020; Cortelli et al., 2013). Due to the high profusion of bacterial exposure in the mouth, the host tissue responds with a protective mechanism that initially leads to the destruction of soft tissues. This initial inflammation on dental implants is termed peri‐implant mucositis; if it is not diagnosed and treated, it may lead to irreversible inflammation that also extends to the hard tissue and develops into peri‐implantitis (Belibasakis et al., 2015; Derks & Tomasi, 2015; L. Heitz‐Mayfield & Salvi, 2018; L. J. Heitz‐Mayfield, 2008; Mikolai et al., 2020; F. Schwarz et al., 2018). Patient home care combined with early clinical diagnosis is a very important element for implant success and maintenance. Mucositis and initial peri‐implantitis can be effectively treated conservatively.

### 1.3 aMMP‐8 and point‐of‐care testing

**Figure 1:**
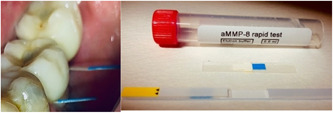
Sample strip placements in implant sulci. Photographs of an active matrix metalloproteinase‐8 point‐of‐care sample collection container, peri‐implant sulcular fluid collection strip, and dipstick test with a reading window.

Clinical observations are required for the diagnosis of dental implant tissue health. Traditionally, clinical evaluation is performed by measuring the BOP, implant pocket depth, and by performing X‐ray evaluations (Berglundh et al., 2018). However, there is an alternative method with which to assess the condition of the tissues around implants. Biomarker‐based aMMP‐8 point‐of‐care testing (POCT) has been extensively studied and is an accurate and validated method (Alassiri et al., 2018; Deng et al., 2021; Golub et al., 2020; Lähteenmäki et al., 2020; Räisänen et al., 2018, 2019; Sorsa, Alassiri, et al., 2020; Ziebolz et al., 2017). The aMMP‐8 test can be used to detect active and early collagenolysis in dental implant tissues. aMMP‐8 is a lateral flow peri‐implant chair‐side/POCT technology validated for periodontal/peri‐implant diagnosis and screening. aMMP‐8 has been successfully implemented as a key biomarker of the new periodontitis/peri‐implantitis classification, and currently, only commercially available point‐of‐care oral fluid biomarker tests are implemented in the new classifications (Alassiri et al., 2018; Deng et al., 2021; Sorsa, Alassiri, et al., 2020; Sorsa, Bacigalupo, et al., 2020; Sorsa et al., 2021). aMMP‐8 POCT is the only Food and Drug Administration (USA) and European Union‐approved peri‐implantitis diagnostic test (Alassiri et al., 2018; Al‐Majid et al., 2018; Sorsa et al., 2016, 2017, 2019). It is a lateral flow immunoassay that detects elevated aMMP‐8 levels. Its sensitivity is 75%–85%, its specificity is 80%–90%, and it is an alternative and adjunctive diagnostic method (Deng et al., 2021; Sorsa, Alassiri, et al., 2020; Sorsa et al., 2021). Compared to the standard evaluation, it strengthens the diagnostic procedure by accounting for disease activity (Golub et al., 2020; Lähteenmäki et al., 2020; Sorsa, Alassiri, et al., 2020; Sorsa, Bacigalupo, et al., 2020). The outcome of the test can be read visually as one (test negative) or two (test positive) lines that emerge in a reading window, similar to the classical point‐of‐care pregnancy test (Figure 1). The results can also be read quantitatively with a device/reader that provides the quantitative and numerical value of the test in 5–7 min.

This study aimed to evaluate the effects of FLJ, used as a mouthwash, on mucositis inflammation in dental implants. The effects were observed and evaluated by recording VPI, BOP, and aMMP‐8 levels.

## 2 MATERIALS AND METHODS

A total of 20 patients (male‐to‐female ratio, 8:12; mean age, 71.7; FLJ group, 75 ± 5.8; control group, 68.3 ± 9.4) were recruited from a private dental clinic (Hammasklinikka Kruunu, Tampere, Finland). Age, smoking habits, and presence of chronic diseases were recorded (Table 1). The participants were divided into two groups to evaluate the effect of FLJ (Pärnänen, 2017; Lingora®, Lingora/Pirjo Pärnänen, Vantaa, Finland) on dental implant attachment tissues through traditional oral clinical investigation methods (BOP and VPI) and an aMMP‐8 chair‐side/POCT. The inclusion criteria were one or more dental implants in the anterior or posterior areas with a screw‐retained crown. The implant brand used was Nobel Biocare® (Nobel Biocare Services AG, Zürich‐Flughafen, Switzerland). The patients underwent maintenance treatment every 6 months. Patients were excluded if they were pregnant, had received antibiotic medications in the last 6 months, or if they had used CHX mouthwash in the last 6 months. No periodontal or peri‐implant treatments were performed at the beginning of or during the trial. The patients used either a manual or an electric toothbrush twice daily. Patients in both were instructed to perform interdental cleaning with interdental brushes approximately once a day using the same toothpaste (Oral‐B Pro‐Expert Professional Protection; Procter & Gamble, Cincinnati, OH, USA) and their normal home care tools for 30 days before, during, and after the lingonberry oral rinse period.

**Table 1:** General characteristics of the patients by group (*n* = 20)

Characteristic	FLJ group (*n* = 10)	Control group (*n* = 10)	*p* Value
Sex			.650
Female	5	7	
Male	5	3	
Patient age (years)			.068
Mean ± SD	75 ± 5.8	68.3 ± 9.4	
Min–max	68–85	56–82	
Smoking			1.000
Yes	1	1	
No	9	9	
Diabetes			1.000
Yes	1	1	
No	9	9	
Heart disease, *n* (%)			1.000
Yes	3	3	
No	7	7	

*Note*: Fisher's exact test was used for sex, smoking, diabetes, and heart disease and a *t* test was used for patient age.

Abbreviation: FLJ, fermented lingonberry juice.

FLJ was used as a mouthwash. Its concentrated, naturally occurring sugars were reduced, and there were no additives. Ten participants (Group 1, FLJ group) used 10 ml of FLJ twice daily (30 s) for 15 days, after which they discontinued the mouthwash for 15 days (washout period). Ten participants (Group 2, control group) did not use mouthwash. According to our previous clinical oral fermented lingonberry mouthwash study, a 15‐day trial length was suitable for this pilot study. The washout period is sufficient as long as the mouthwash period enables observation of the clearance of the treatment effect (Pärnänen et al., 2019).

Oral clinical investigation and diagnoses were performed by one dentist at each time point, including VPI (scale ranging from VPI (0) = *no plaque up to VPI*; (3) = *plaque on all tooth surfaces*). BOP (yes/no) was recorded using a standard millimeter‐graded American Eagle WHO probe (American Eagle Manufacturing Co., New Bern, NC, USA: AEEP23/WHOBX) around the implants. The periodontal and peri‐implant diagnoses of the patients by group (*n*= 20) are shown in Table 2. Peri‐implant health was defined as the absence of ≥4‐mm probing depths, BOP, erythema, swelling, and suppuration around the implant without radiographic bone loss. Peri‐implant mucositis was defined as the presence of BOP with or without erythema, swelling, and/or suppuration around the implant and without radiographic bone loss. Peri‐implantitis was defined as the presence of ≥4‐mm probing depths and BOP with or without erythema, swelling, and/or suppuration around the implant, together with radiographic bone loss ≥2 mm (Berglundh et al., 2018).

**Table 2:** Periodontal status and peri‐implant diagnoses of the participants and their association as calculated using Fisher's exact test for the FLJ and control groups

Characteristics	Healthy	Mucositis	Peri‐implantitis	*p* Value
FLJ group	0	4	6	.650
Control group	2	3	5	

*Note*: Peri‐implant health was defined as the absence of ≥4‐mm probing depths, BOP, erythema, and swelling and suppuration around the implant, all without radiographic bone loss. Peri‐implant mucositis was defined as the presence of BOP with or without erythema, swelling, and/or suppuration around the implant and without radiographic bone loss. Peri‐implantitis was defined as the presence of ≥4‐mm probing depths and BOP with or without erythema, swelling, and/or suppuration around the implant, together with radiographic bone loss ≥2 mm (Berglundh et al., 2018).

Abbreviations: BOP, bleeding on probing; FLJ, fermented lingonberry juice.

At the same time points as above, an additional sulcus fluid rinse was collected for chair‐side aMMP‐8 POC tests (ImplantSafe®/ORALyzer®; Dentognostics, Jena, Germany), as described in the study by Lähteenmäki et al. (2020). aMMP‐8 quantitative online evaluation was performed after 0, 15, and 30 days according to the manufacturer's instructions. The sampling site was prepared by removing excess saliva using a short blast of air and water. A sterile peri‐implant sulcular fluid (PISF) collection test strip was placed in the sulcus at the sampling site using tweezers for 30 s, after which the collection strip was immersed in the elution fluid of the test kit for 5 min. The dipstick test was immersed in the elution fluid for 15 s, and then removed and placed in the compartment of the ORALyzer® reader. After 5 min, the aMMP‐8 level results appeared in the result window of the reader, and at the same time, the qualitative sample analysis was visible from the dipstick: two blue lines identified a positive test (aMMP‐8 level ≥ 20 ng/ml), and one blue line, as the test control, indicated a negative test (aMMP‐8 level ≤ 20 ng/ml). The results of the aMMP‐8 POC/chair‐side enzyme test were documented using a photograph after the test result was visible. The patients were treated at the end of the study period with ultrasonic implant debridement, scaling, and root planning, and an airflow powder protocol (EMS®, EMS, Nyon, Switzerland) by an experienced oral hygienist.

The study was approved by the ethical committees of the Stockholm Community, Sweden (2016/1410‐31) and Helsinki University Central Hospital, Finland (1271/2019 and 51/13/02/2009). The procedures were undertaken with the understanding and written consent of each subject and according to ethical principles, including the World Medical Association Declaration of Helsinki.

### 2.1 Statistical analysis

#### 2.1.1 Sample size estimation

The sample size was estimated based on a correlation of 0.65 among repeated measures for the mixed design repeated measures analysis of variance (ANOVA). The correlation was estimated to be somewhat high, as patients’ aMMP‐8, VPI, and BOP measurements should be quite consistent across time and as no interference in the form of anti‐infective treatment was planned for patients during their visits to the dental hygienist. A total of 20 patients were required to achieve a power of 80.9% with a medium effect size and a significance level of .05. Thus, with 10 patients in the FLJ group and 10 patients in the control group, there was an 80.9% chance of correctly rejecting the null hypothesis of no significant effect of the interaction.

**Figure 2:**
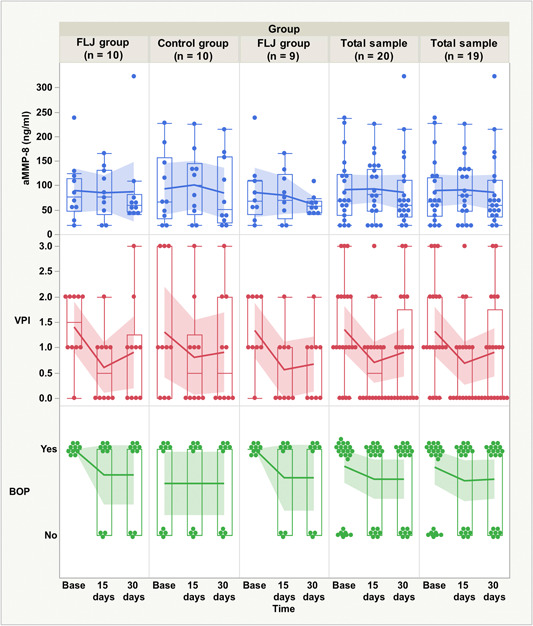
Boxplots of active matrix metalloprotease‐8 (aMMP‐8, ng/ml), visible plaque index (VPI), and bleeding on probing (BOP) data among patients in the fermented lingonberry juice (FLJ) mouthwash and control groups as well as for both groups together (with and without a patient with outlier values) at three time points (at baseline, 15 days, and 30 days). The line represents the group mean with a 95% confidence interval.

The general patient characteristics are summarized in Table 1. Differences in categorical and continuous variables between the FLJ and control groups were analyzed using Fisher's exact test and a *t* test, respectively. The effects of the FLJ mouthwash on aMMP‐8 levels, VPI, and BOP were measured in the FLJ and control groups (at baseline, 15 days, and 30 days) (Table 3). The differences in the mean levels of aMMP‐8, VPI, and BOP were investigated using a mixed‐design repeated‐measures ANOVA analysis (Figure 2). To adjust for multiple comparisons, the Bonferroni correction was used for pairwise comparisons in the repeated‐measures ANOVA. One patient in the FLJ group had outlier aMMP‐8 (extreme outlier, value above Q3 + 3 × interquartile range [IQR]) and VPI (outlier, value above Q3 + 1.5 × IQR) measurements at the third time point (30 days), which were considered in the statistical calculations (with and without this patient). Statistical significance was defined as a two‐tailed *p* < .05. All statistical calculations were performed using SPSS Statistics version 27 (IBM Corp) for Macintosh and the rstatix package (version 0.6.0) in R statistical software version 3.6.3. Figures were created using JMP Pro version 15 (SAS Institute Inc., Cary, NC, USA) for Macintosh software.

**Table 3:** Results of intra‐ and intergroup comparisons of aMMP‐8, VPI, and BOP at baseline, after 15 days of mouthwash use, and after a 15‐day washout period

	Model 1: All patients			Model 2: A patient with outlier values excluded		
Variable	Group	Mean (95% CI)	*p* Value	Group	Mean (95% CI)	*p* Value
aMMP‐8 at baseline	FLJ (*n* = 10)	89.23 (63.19)	.888 (Time)	FLJ (*n* = 9)	85.32 (34.80–135.83)	.341 (Time)
	Control (*n* = 10)	92.66 (40.00–145.32)	.826 (Time × Group)	Control (*n* = 10)	92.66 (40.00–145.32)	.809 (Time × Group)
aMMP‐8 at 15 days	FLJ (*n* = 10)	84.63 (48.86–120.40)		FLJ (*n* = 9)	79.58 (40.95–118.22)	
	Control (*n* = 10)	100.70 (51.37–150.03)		Control (*n* = 10)	100.70 (51.37–150.03)	
aMMP‐8 at 30 days	FLJ (*n* = 10)	86.80 (25.85–147.75)		FLJ (*n* = 9)	60.59 (44.52–76.65)	
	Control (*n* = 10)	84.12 (32.38–135.87)		Control (*n* = 10)	84.12 (32.38–135.87)	
VPI at baseline	FLJ (*n* = 10)	1.40 (0.90–1.90)	.002 (Time)	FLJ (*n* = 9)	1.33 (0.79–1.88)	.003 (Time)
	Control (*n* = 10)	1.30 (0.40–2.20)	.677 (Time × Group)	Control (*n* = 10)	1.30 (0.40–2.20)	.584 (Time × Group)
VPI at 15 days	FLJ (*n* = 10)	0.60 (0.10–1.10)	*t*1–*t*2, *p* = .007	FLJ (*n* = 9)	0.56 (0.00–1.11)	*t*1–*t*2, *p* = .013
	Control (*n* = 10)	0.80 (0.06–1.54)	Bonferroni	Control (*n* = 10)	0.80 (0.06–1.54)	*t*1–*t*3, *p* = .026 Bonferroni
VPI at 30 days	FLJ (*n* = 10)	0.90 (0.19–1.61)		FLJ (*n* = 9)	0.67 (0.12–1.21)	
	Control (*n* = 10)	0.90 (0.11–1.69)		Control (*n* = 10)	0.90 (0.11–1.69)	
BOP at baseline	FLJ (*n* = 10)	1.00 (1.00–1.00)	.378 (Time)	FLJ (*n* = 9)	1.00 (1.00–1.00)	.339 (Time)
	Control (*n* = 10)	0.60 (0.23–0.97)	.378 (Time × Group)	Control (*n* = 10)	0.60 (0.23–0.97)	.339 (Time × Group)
BOP at 15 days	FLJ (*n* = 10)	0.70 (0.35–1.05)		FLJ (*n* = 9)	0.67 (0.28–1.05)	
	Control (*n* = 10)	0.60 (0.23–0.97)		Control (*n* = 10)	0.60 (0.23–0.97)	
BOP at 30 days	FLJ (*n* = 10)	0.70 (0.35–1.05)		FLJ (*n* = 9)	0.67 (0.28–1.05)	
	Control (*n* = 10)	0.60 (0.23–0.97)		Control (*n* = 10)	0.60 (0.23–0.97)	

*Note*: Model 1 and 2 comparisons: Model 1 represents all patients without outliers; in Model 2, one extreme outlier was excluded.

Abbreviations: aMMP‐8, active matrix metalloproteinase 8; BOP, bleeding on probing; CI, confidence interval; FLJ, fermented lingonberry juice; VPI, visible plaque index.

## 3 RESULTS

The patient characteristics are presented in Table 1. Fifty percent of the participants in the FLJ group were men, and 30% were men in the control group (*p* = .650). The mean ages of the patients were 75.1 and 68.3 years in the FLJ and control groups, respectively (*p* = .068). There were no significant differences in the prevalence of smoking or comorbidities between the FLJ and control groups.

The aMMP‐8, VPI, and BOP measurements for the FLJ and control groups are presented in Figure 2. Positive effects were observed in the FLJ group after the first 15 days when the lingonberry mouthwash was used. There was a decrease in the mean aMMP‐8, VPI, and BOP levels between the first two time points (at baseline and 15 days) in the FLJ group. When one FLJ group patient with outlier values was excluded, the decrease in these three variables (Figure 2) was more pronounced. Similar results between these two time points were observed for the mean VPI but not for aMMP‐8 or BOP in the control group (Figure 2). Second, after the second time point, when both groups were not using the lingonberry mouthwash, the decreasing trend in the periodontal parameters was not as positive as it was during the first 15 days (Figure 2). The mean VPI increased in both groups, but the mean BOP remained unchanged. The effects of the extreme outliers could be seen in the aMMP‐8 and VPI values among the final 15 days in the FLJ group (Figure 2). When outliers were excluded, the mean aMMP‐8 continued to decrease, whereas the mean VPI was smaller. In the control group, there was a small decrease in the mean aMMP‐8 level after a small increase during the first 15 days.

The results of the statistical comparisons are presented in Table 3. However, there was no significant combined effect of time and group on aMMP‐8 (*p* = .826, *n* = 20; *p* = .809, *n* = 19), VPI (*p* = .677, *n* = 20; *p* = .584, *n* = 19), or BOP (*p* = .378, *n* = 20; *p* = .339, *n* = 19). Only the main effect of time was significant for VPI (*p* = .002, *n* = 20; *p* = .003, *n* = 19), which showed a significant difference between the first two time points (at baseline and 15 days; *p* = .007, *n* = 20 and *p* = .013, *n* = 19) and the first and last time points (at baseline and 30 days; *p* = .026, *n* = 19). For aMMP‐8 and BOP, the main effect of time was not significant (*p* = .888, *n* = 20; *p* = .341, *n* = 19; *p* = .378, *n* = 20; *p* = .339, *n* = 19, respectively).

## 4 DISCUSSION

This study was the first of its kind to examine the effects of FLJ on parameters that reflect the course of peri‐implant health and disease using indices and to additionally assess disease activity using the aMMP‐8 POCT. In the current study, 15 days of using the FLJ mouthwash seemed to have similar decreasing effects on PISF aMMP‐8, visible plaque, and BOP levels around dental implants. In the control group, a similar, although smaller, decrease was seen in visible plaque levels, but not in aMMP‐8 or BOP levels, after the first 15 days. Statistically significant differences in the mean aMMP‐8, VPI, and BOP levels between the FLJ and control groups were not observed in this study. A previous clinical study (Pärnänen et al., 2019) demonstrated that FLJ mouthwash decreased whole‐mouth aMMP‐8, BOP, and VPI measured by traditional periodontal techniques in patients with natural teeth. This suggests that the FLJ mouthwash period of only 15 days may have been too short a period of time to achieve site‐specific effects instead of whole‐mouth effects. This is supported by the findings of this study where the differences in aMMP‐8, VPI, and BOP between the groups diminished after the lingonberry regimen ended. Furthermore, exclusion of the effect of outliers revealed that aMMP‐8 levels continued to decrease in the FLJ group, even after discontinuation of the mouthwash regimen. This suggests that the effect of the FLJ mouthwash could be seen earlier in the visible plaque levels, while the antiproteolytic effects lasted longer or were delayed for aMMP‐8 levels that are known to indicate and reflect the risk of ongoing collagenolytic peri‐implant tissue destruction (Lähteenmäki et al., 2020; Pärnänen et al., 2020).

There were certain limitations in this study that may explain why the results did not reach statistically significant levels, even though the trend of the parameter values decreased during the FLJ mouthwash study period. Due to the relatively short study period, visits to the dental hygienist may have influenced the results by motivating some study patients in both groups toward better oral hygiene than normal. This might have decreased the difference between the two groups. Therefore, in order to achieve sufficient estimate precision, it seems essential to control for this kind of confounding factor more carefully in future studies with longer study periods and larger sample sizes. However, it is unclear whether the toothpaste used by all patients during the study period had any effect on the results. To our knowledge, there is no clear evidence in the literature demonstrating that toothpaste exhibits scientifically relevant oral anti‐inflammatory actions. As expected, the participants used toothpaste during the FLJ and washout periods. Thus, the potential antimicrobial and anti‐biofilm effects of the toothpaste remained a constant factor between both groups in this study, which did not cause confounding between the groups. It is worth noting that toothpaste is part of the recommended oral hygiene regime worldwide, making it difficult to exclude its potential anti‐inflammatory effects in study designs.

The treatment of peri‐implant mucositis, especially peri‐implantitis, is challenging. The current study was a clinical pilot study to evaluate the effects of a natural substance as an alternative adjunctive therapy for these conditions. Further studies with larger patient cohorts and significantly longer study periods are required to verify and extend the results of the present study. FLJ appears to be a promising and safe aid in oral home care for reducing plaque levels, gingival bleeding, and inflammation around dental implants and throughout the mouth. Lingonberries are known to have anti‐inflammatory, antioxidant (radical oxygen scavenging), antiproteolytic, and antimicrobial properties (Pärnänen et al., 2021). FLJ does not inhibit *Lactobacilli* growth, and according to Cereda et al. (2018), probiotic *Lactobacilli* have also been proposed to have beneficial effects on chemoradiotherapy‐related oral mucositis and peri‐implant mucositis. Currently, CHX is regarded as the gold standard for the treatment of oral and implant‐related infections. However, there are indications that repeated exposure to the most commonly used antiseptics, such as CHX, may induce resistance to oral bacteria in deep biofilms, which has never been demonstrated for lingonberries (S. R. Schwarz et al., 2021).

## 5 CONCLUSION

This study was the first of its kind to examine the effects of FLJ on the parameters that reflect dental implant health and disease using indices and to additionally assess the collagenase activity in PISF using the aMMP‐8 POCT. aMMP‐8 values reflect early and developing collagenolytic inflammation in the oral cavity. When analyzed in the PISF, these values are exact site‐specific indicators and biomarkers of the progression of peri‐implantitis and may be used to monitor the oral effects of FLJ. The continuous reductions in the amount of plaque, aMMP‐8, and BOP by FLJ rinsing were promising, considering the relatively short study period. This pilot study provides a promising basis for large‐scale studies with significantly longer study periods to verify and extend these results in the future.

## AUTHOR CONTRIBUTIONS

Hanna Lähteenmäki performed the clinical examinations and sample gathering. Ismo T. Räisänen and Hanna Lähteenmäki performed statistical analyses. All authors contributed to the study design, writing, and manuscript review.

## CONFLICTS OF INTEREST

Prof. Timo Sorsa is the inventor of US patents 5652223, 5736341, 5866432, 6143476, 20170023571A1 (Grant no. 6.6.2019), WO 2018/060553A1 (Grant no. 31.5.2018), a coinventor of the 10488415 B2 patent, the Japanese patent 2016‐554676, and the South Korean patent 10‐2016‐7025378. Dr. Pirjo Pärnänen is the inventor of patent EP 2585087B1 and the holder of the trademark Lingora®.

## Data Availability

The data that support the findings of this study are available from the corresponding author upon reasonable request.
